# Ultrafast electronic and vibrational dynamics in brominated aluminum corroles: Energy relaxation and triplet formation

**DOI:** 10.1063/1.4949363

**Published:** 2016-05-12

**Authors:** T. Stensitzki, Y. Yang, A. Berg, A. Mahammed, Z. Gross, K. Heyne

**Affiliations:** 1 Institute of Experimental Physics, Free University Berlin, Arnimallee 14, 14195 Berlin, Germany; 2 Institute of Chemistry, The Hebrew University of Jerusalem, Jerusalem 91904, Israel; 3 Technion-Israel Institute of Technology, Schulich Faculty of Chemistry, Haifa 32000, Israel

## Abstract

We combined femtosecond (fs) VIS pump–IR probe spectroscopy with fs VIS pump–supercontinuum probe spectroscopy to characterize the photoreaction of the hexacoordinated Al(tpfc-Br_8_)(py)_2_ in a comprehensive way. Upon fs excitation at ∼400 nm in the Soret band, the excitation energy relaxes with a time constant of (250 ± 80) fs to the S_2_ and S_1_ electronic excited states. This is evident from the rise time of the stimulated emission signal in the visible spectral range. On the same time scale, narrowing of broad infrared signals in the C=C stretching region around 1500 cm^−1^ is observed. Energy redistribution processes are visible in the vibrational and electronic dynamics with time constants between ∼2 ps and ∼20 ps. Triplet formation is detected with a time constant of (95 ± 3) ps. This is tracked by the complete loss of stimulated emission. Electronic transition of the emerging triplet absorption band overlaps considerably with the singlet excited state absorption. In contrast, two well separated vibrational marker bands for triplet formation were identified at 1477 cm^−1^ and at 1508 cm^−1^. These marker bands allow a precise identification of triplet dynamics in corrole systems.

## I. INTRODUCTION

Corroles are developing class of photosensitizers with significant chemical and photophysical properties and relatively unexplored potential.^1,2^ The simple and efficient procedure of corroles synthesis combined with readily tuned physical and chemical characteristics by varying the peripheral substituents,^3–6^ central metal,^5,7^ and axial ligands^8,9^ has revived substantial interest in employing these contracted porphyrinoids in various fields. Examples for application of the corroles include dye-sensitized solar cells,^7,10^ photodynamic therapy,^11–13^ photodynamic detection,^14^ photodynamic inactivation of mold fungi and green algae,^15,16^ regular and sophisticated optical imaging,^17,18^ formation of singlet oxygen for catalysis,^19–21^ and corrole-based electron and energy transfer systems.^22–24^ In this context, the ability to control the corrole's parameters such as fluorescence,^6,25^ phosphorescence,^26–29^ and singlet oxygen quantum yield,^30^ energy,^31^ and lifetime of their photoexcited states^26,32–34^ is fundamental in optimizing the corrole based photocatalysts for their specific application.^35^ However, there are very few in-depth reports on the physical and spectroscopic features of corroles. The most striking example is vibrational spectroscopy (IR, RR, and more sophisticated methods), very well established for porphyrins^36–39^ but very limited for corroles.^40–43^ What is more, there is only one prior publication that focused on the ultrafast vibrational processes involved therein.^44^ A recent study on other corrole systems investigated electronic dynamics from femtoseconds to microseconds, but a separation of singlet and triplet dynamics on the short time scale remained difficult.^45^ An improved insight into the fundamental properties of post-transition metallocorroles is hence clearly required for learning how to utilize them in potentially practical applications.

## II. RESULTS AND DISCUSSION

**FIG. 1.**
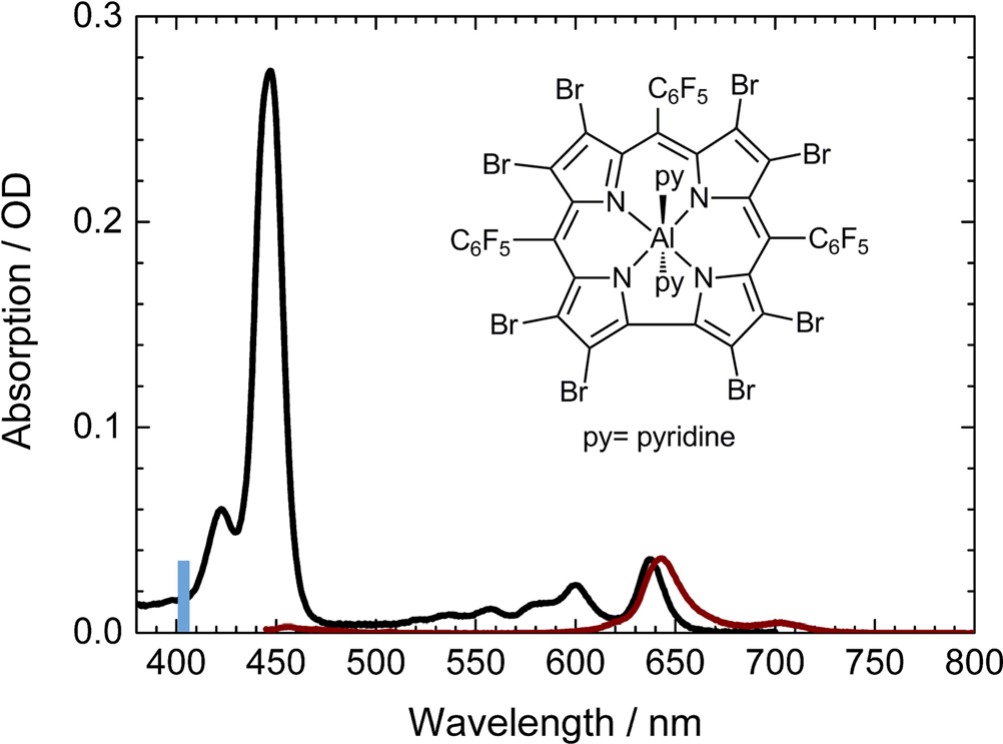
Absorption spectrum (black) and emission spectrum (wine) of Al(tpfc-Br_8_)(py)_2_; excitation wavelength is indicated by a blue bar at 403 nm; inset: Molecular structure of Al(tpfc-Br_8_)(py)_2_.

In this study, we investigated hexacoordinated aluminum(III) 2,3,7,8,12,13,17,18-octabromo-5,10,15-tris(pentafluorophenyl) corrole, Al(tpfc-Br_8_)(py)_2_, upon excitation at the high energy side of the Soret band at 400 nm. The absorption spectrum exhibits a Soret band maximum at 445 nm, and two maxima in the Q band at 600 nm and 637 nm, resulting from the S_0_ → S_2_ transition and S_0_ → S_1_ transition.^44^ Fluorescence maxima were observed at 643 nm and 704 nm, as depicted in Fig. 1. Two pyridine ligands are located above and below the corrole plane, directly interacting with the aluminum (see Fig. 1 inset).

**FIG. 2.**
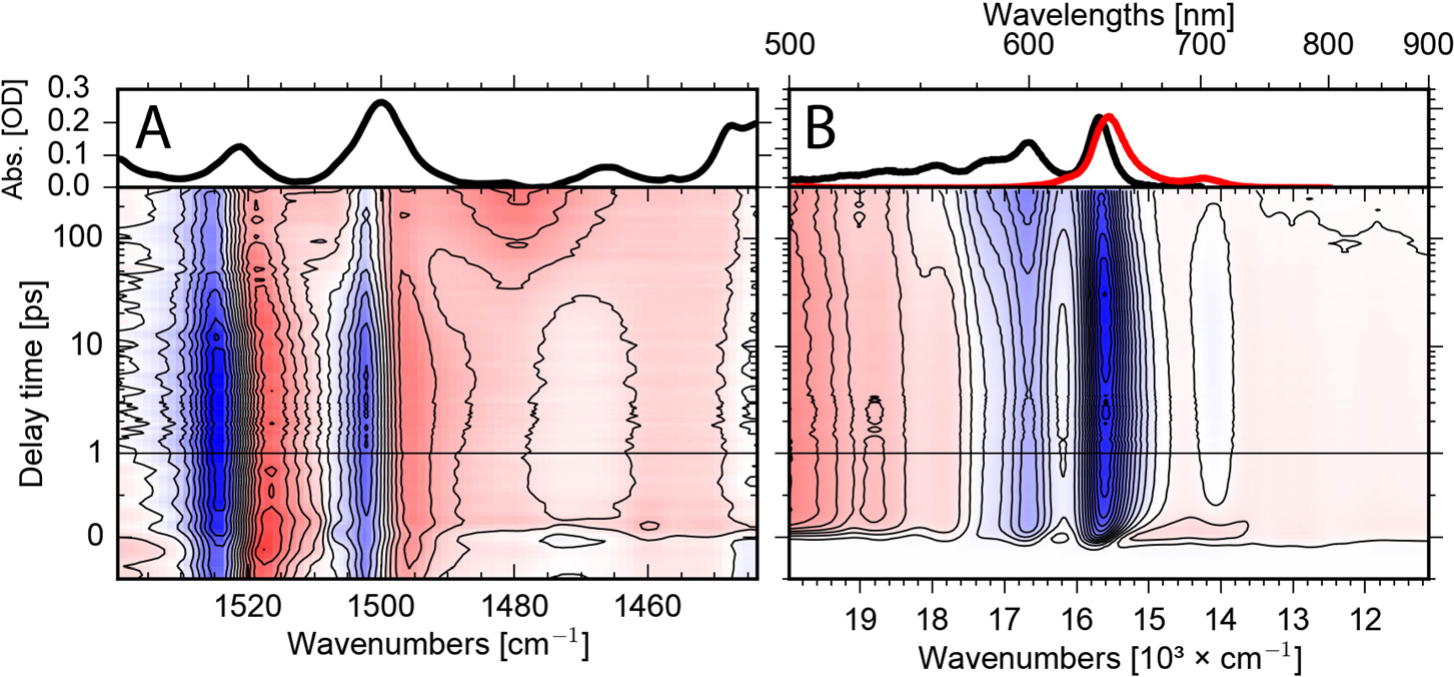
A. Upper panel: Infrared absorption spectrum of Al(tpfc-Br_8_)(py)_2_ in the investigated spectral region. Lower panel: 2D map of the vibrational absorbance change upon excitation at ∼400 nm as a function of wavenumber and delay time. Negative signals (blue) indicate bleaching signals, while positive signals (red) are due to singlet excited state absorption and triplet absorption. B. Upper panel: Visible absorption spectrum of Al(tpfc-Br_8_)(py)_2_ (black line) and fluorescence signal (red line) in the investigated spectral region. Lower panel: 2D map of the absorbance change as a function of wavelengths and pump-probe delay time. Excitation at the same wavelength ∼400 nm. Positive signals (red) show excited state and triplet absorption; negative signals (blue) indicate bleaching and stimulated emission signals. The transient changes of both experiments can be compared directly.

**FIG. 3.**
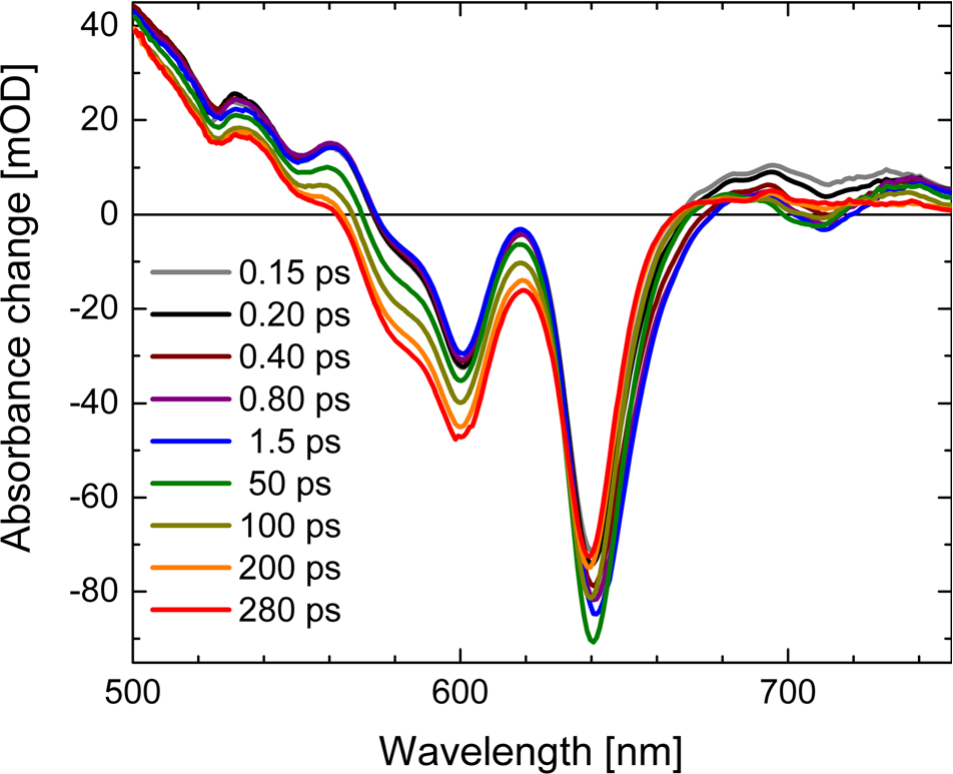
Absorbance changes of Al(tpfc-Br_8_)(py)_2_ as a function of wavelength at different pump-probe delay times. Positive signals indicate increased absorption upon excitation at ∼400 nm, while negative signals are due to reduced absorption.

The electronic dynamics were studied by femtosecond VIS pump–supercontinuum probe spectroscopy on a time scale from femtoseconds to 280 ps. We investigated the dynamics in a broad spectral range from 500 nm to 975 nm with a system response of ∼100 fs. Figure 2(b) presents the difference absorbance change upon photoexcitation in a 2D map. Bleaching and stimulated emission signals (negative) are shown in blue, while increased absorption due to excited state and product bands as triplet absorption are colored in red. As displayed in Fig. 2(b), the positive signal from 500 nm to 574 nm, the positive signal from 670 nm to 975 nm, as well as the negative signal from 574 nm to 670 nm appear instantaneously upon excitation. We assign the positive signal to singlet excited state absorption, and the negative signal to bleaching absorption of the ground state. On a time scale of a few hundred femtoseconds, negative signals rise at the position of the fluorescence at around 650 nm and around 710 nm (see also Fig. 3). We assign these signals to stimulated emission. On a longer time scale of 100 ps, these stimulated emission signals vanish completely, indicating the decay of the electronic excited singlet state. On the same time scale, the bleaching signal from 570 nm to 625 nm increases, due to the loss of the singlet excited state absorption in this spectral range. This is well visible in Fig. 3 for long delay times. This demonstrates the triplet formation on a time scale of 100 ps, since the bleaching signal does not recover, while the stimulated emission signal, reflecting singlet excited state population, vanishes completely.

In Fig. 3, we display the absorbance difference spectra as a function of wavelengths at different delay times. The complete loss of stimulated emission signals around 650 nm and around 710 nm is clearly visible, enabling a definite assignment of the triplet state. Around 600 nm, a strong effect of the decaying singlet excited state absorption is displayed; while for wavelengths longer than ∼670 nm and for short wavelength around 500 nm, a significant positive signal remains. We attribute this remaining positive signal to triplet absorption. Triplet generation in Br_8_Al(tpfc)(py)_2_ was previously observed by the time resolved EPR studies.^5^ Importantly, the spectral shape of singlet excited state absorption and triplet absorption are strongly overlapping, and thus difficult to separate.

**FIG. 4.**
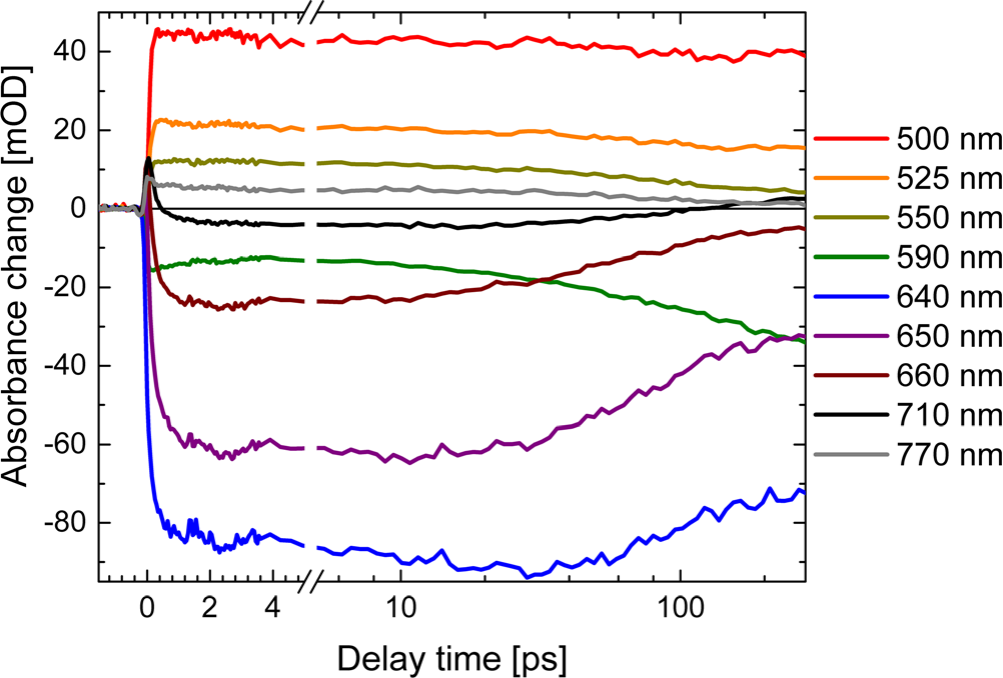
Transients of the visible dataset upon excitation of Al(tpfc-Br_8_)(py)_2_ at ∼400 nm (thick solid lines). Simulations of the transients are presented by thin lines.

Transients at selected wavelengths are presented in Fig. 4. At short wavelengths around 500 nm (red line, Fig. 4), the transient absorbance change is minor, because the loss of singlet excited state signal around 100 ps is compensated by the newly generated triplet signal on the same time scale. This indicates a reaction pathway from the singlet excited state to the triplet state. Transients at 550 nm (dark yellow line) and 590 nm (green line) in Fig. 4 illustrate the increase of the negative bleaching contribution on a time scale of ∼100 ps, due to the loss of singlet excited state absorption. In this spectral range, the triplet absorption is smaller than the singlet excited state absorption and cannot compensate the loss of positive signal anymore. At wavelengths of 640 nm (blue line), 650 nm (purple line), 660 nm (wine line), and 710 nm (black line), the negative signal rises within a few hundred femtoseconds, stays nearly constant, and decays on a time scale of ∼100 ps. The transient at 770 nm (grey line) in Fig. 4 exhibits an instantaneous rise to a positive signal due to the singlet excited state absorption that decays on a time scale of ∼100 ps to a smaller positive signal. The remaining positive signal is not vanishing, reflecting triplet absorption.

**FIG. 5.**
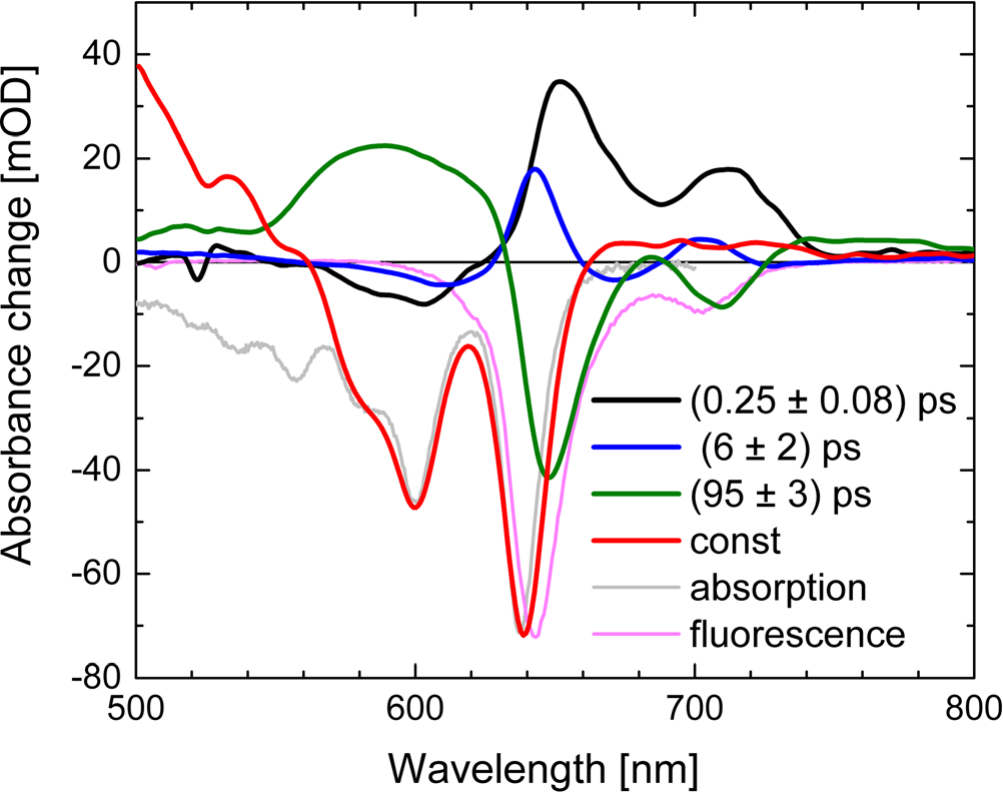
Decay associated spectra (DAS) of the visible dataset upon excitation of Al(tpfc-Br_8_)(py)_2_ at ∼400 nm. Absorption (grey line) and fluorescence (pink line) are scaled and plotted for comparison.

Global simulation of the dataset with a multi exponential approach results in three relevant time constants of τ_1_ = (0.25 ± 0.08) ps, τ_2_ = (6 ± 2) ps, τ_3_ = (95 ± 3) ps, and a constant τ_4_. The decay associated spectra (DAS) for these time constants are shown in Fig. 5. Here, we can use a true sequential model with a sequence of several steps. Upon excitation, the singlet excited state in the Soret band is formed instantaneously, followed by transfer to the singlet excited state of the Q band accompanied with the rise of stimulated emission with 250 fs. On the picosecond time scale, energy relaxation processes occur in the singlet excited state, followed by a complete singlet excited state decay into the triplet manifold with 95 ps. The constant component in Figure 5 reflects the difference between ground state bleaching and triplet absorption. As a result of the sequential model, the presented decay associated spectra (DAS) in Figure 5 show the decay and rise of intermediate states with the given time constants.

The DAS of τ_1_ (DAS_1_) exhibits positive signals at the spectral positions of the fluorescence maxima, and small negative signals around 600 nm. This demonstrates that the stimulated emission rises with a time constant τ_1_, and probably small excited state absorption increases around 600 nm. The DAS_2_ exhibits a derivative like pattern with positive/negative contributions at 609 nm (−)/643 nm (+) and 674 nm (−)/702 nm (+) and a time constant of about 6 ps (blue line Fig. 5). This can be interpreted as cooling of vibrational modes coupled to the singlet excited state absorption, resulting in spectral shifts and narrowing of the stimulated emission. Extending the global fit to five exponentials results in splitting of DAS_2_ in two contributions. This is presented in Fig. S1 with two time constants of 2 ps and 18 ps for cooling.^50^ DAS_3_ has negative signals at spectral positions of the fluorescence, significant positive signals around 600 nm, and small positive signals around 500 nm and 800 nm. We assign time constant τ_3_ to the singlet excited state decay and triplet formation. Positive signals indicate decay of the singlet electronic excited state signal that is not compensated by the triplet absorption, and negative signals indicate the decay of the stimulated emission signal. Note that the stimulated emission signal vanishes completely, best visible at around 700 nm.

Since the stimulated emission and the electronic absorption spectra of excited state, triplet state, and ground state provide considerable spectral overlap, we decided to investigate the vibrational marker band region around 1500 cm^−1^ after excitation at ∼400 nm.^44^

The absorbance change upon excitation as a function of wavenumber and delay time is presented as a 2D map in Fig. 2(a) lower panel. Observed are pronounced bleaching signals at 1501 cm^−1^ and 1523 cm^−1^, matching the positions of the absorption bands in Fig. 2(a) upper panel. These bleaching signals were assigned to C=C stretching vibrations ν(C=C)_1_ and ν(C=C)_2_ at 1501 cm^−1^ and 1523 cm^−1^, respectively. The bleaching band around 1470 cm^−1^ in Fig. 2(a) is indicated by a small perturbed free induction decay signal^44^ at delay times before time zero in Fig. 2(a). This negative signal is masked by positive contributions for all detected delay times up to 300 ps. Upon excitation, we observe strong positive signals red-shifted to the bleaching bands at 1501 cm^−1^ and 1523 cm^−1^ due to the vibrations in the singlet electronic excited state. We assign these vibrations to ν(C=C)_1_* and ν(C=C)_2_* vibrations in the S_1_ electronic excited state. On a time scale of a few hundred femtoseconds, the positive bands show a significant broadening to lower energy frequencies. We assign this feature to excitation of higher low-frequency vibrational states in the S_1_, i.e., a hot population of the observed vibrations due to fast energy redistribution of the excess energy. A striking feature in Fig. 2(a) is the decay of the ν(C=C)_1_* and ν(C=C)_2_* signals on a time scale of 100 ps, exactly the time scale when stimulated emission signal vanishes in Fig. 2(b). Accompanied with this decay, new positive signals rise at 1480 cm^−1^ and 1506 cm^−1^. We assign the decay of the ν(C=C)_1_* and ν(C=C)_2_* absorption to a decay of the singlet excited state, and the rise of the positive signals at 1480 cm^−1^ and 1506 cm^−1^ to triplet formation. Hence, the new emerging bands at 1480 cm^−1^ and 1506 cm^−1^ represent the marker bands for a triplet state in Al(tpfc-Br_8_)(py)_2_.

**FIG. 6.**
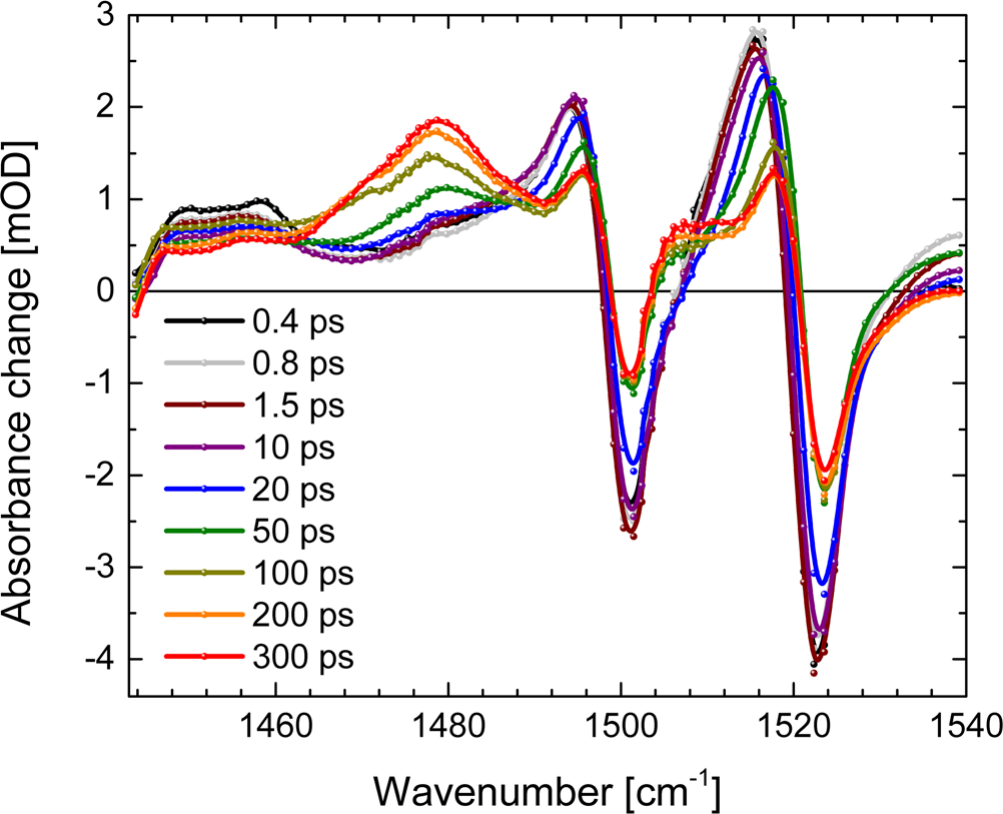
Vibrational absorbance difference spectra of Al(tpfc-Br_8_)(py)_2_ as a function of wavenumber at different delay times. Positive signals are for increased absorption and negative signals for decreased absorption (bleaching bands).

In Fig. 6, the absorbance difference spectra at different delay times are presented. At early delay times, no signatures indicative of the triplet marker bands are visible. The positive signal around 1540 cm^−1^ exhibits a decay within a few picoseconds. This signal could reflect some population persisting longer in the electronic excited state of the Soret band, resulting in a strongly altered C=C stretching frequency. The early delay times from a few ps to about 30 ps are dominated by narrowing of the positive bands. The cooling effect is also visible by the spectral shift of the zero-crossings on the low energy side of the bleaching signals at 1499 cm^−1^ and 1520 cm^−1^. These spectral shifts presented in Fig. S2 can be simulated with time constants of (1.4 ± 0.3) ps and (19 ± 3) ps.^50^ This is corroborated by spectral shift of the bleaching and stimulated emission band around 650 nm (see Fig. S2) with the time constants of (1.2 ± 0.8) ps and (22 ± 2) ps.^50^ The difference spectrum at 300 ps (red dots and line) in Fig. 6 mainly shows triplet absorption (positive signals) and bleaching bands (negative signals).

**FIG. 7.**
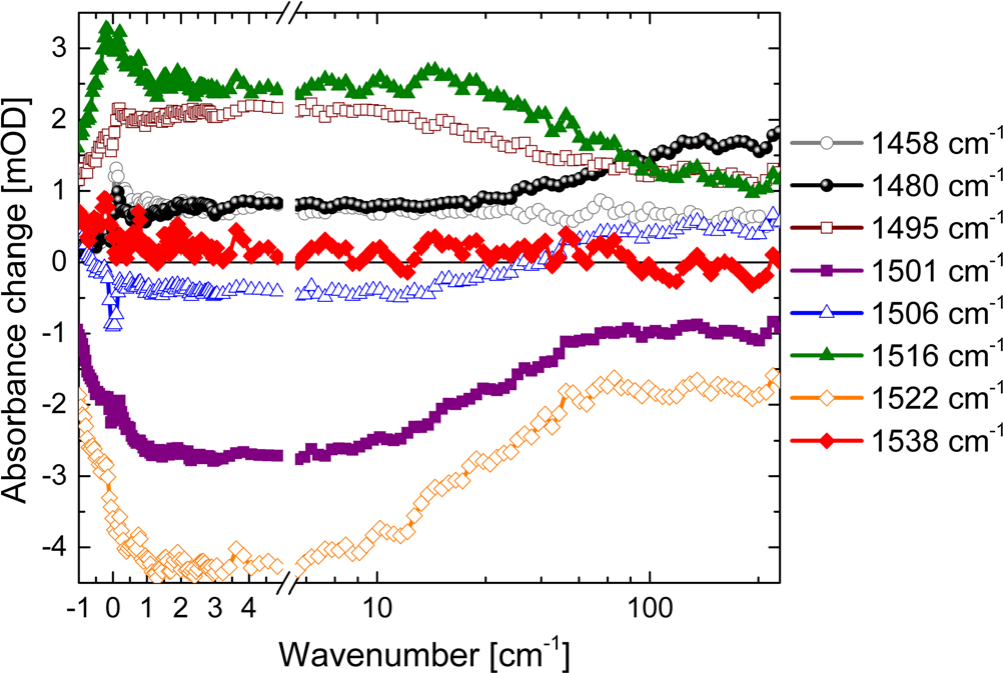
Vibrational transient data at selected wavenumbers as a function of delay time upon excitation of Al(tpfc-Br_8_)(py)_2_ at ∼400 nm.

Fig. 7 presents the transients at selected wavenumbers: At 1516 cm^−1^ (green triangles) and 1522 cm^−1^ (yellow circles), a significant part of the dynamic changes in the sub picosecond time scale. On a time scale of about 20 ps positive/negative absorption pairs decay at 1495 cm^−1^ (+)/1501 cm^−1^ (−) and 1516 cm^−1^ (+)/1522 cm^−1^ (−). Positive absorption of the transients at 1480 cm^−1^ (black dots) and 1506 cm^−1^ (blue triangles) clearly increases around 100 ps, matching the triplet rise time of 95 ps in the visible data (Fig. 5).

**FIG. 8.**
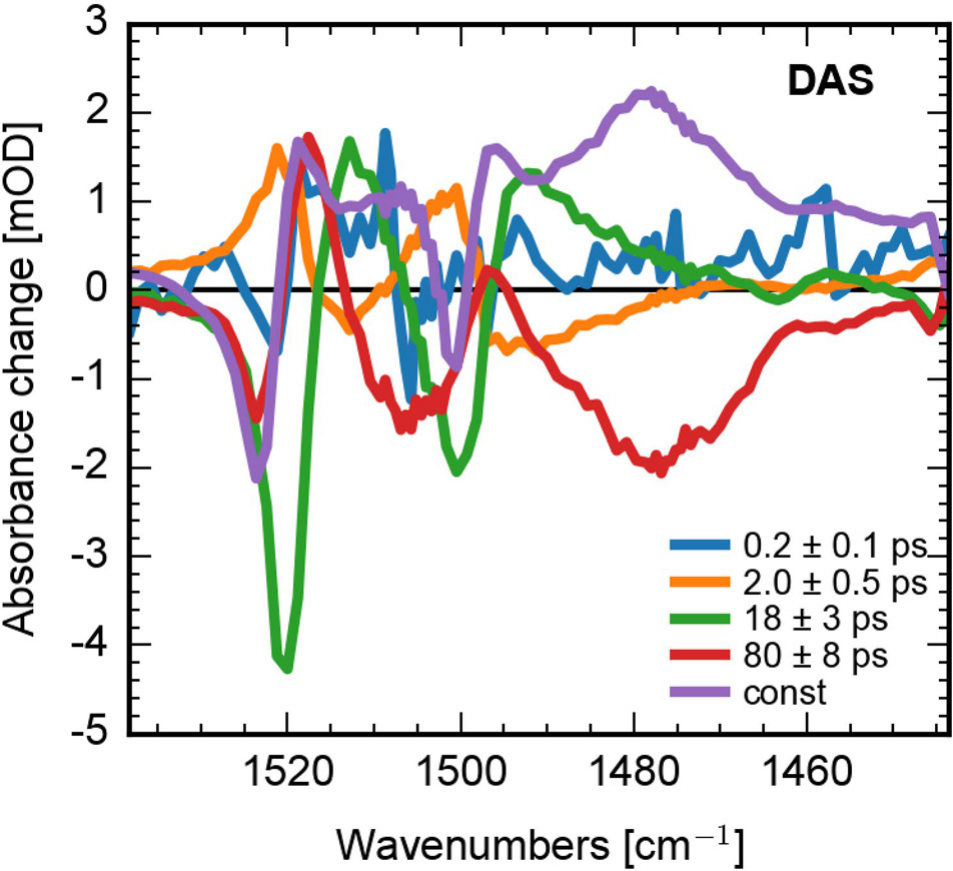
Decay associated spectra of the vibrational dynamics of Al(tpfc-Br_8_)(py)_2_ upon excitation at ∼400 nm. Our true sequential model allows to interpret the spectra as spectra of intermediate states increasing/decaying with the given time constant.

In Fig. 8, we present DAS of the global fit of the vibrational data. We found at least five components to simulate the data. The fast component with (200 ± 100) fs decay time reflects signal decay around 1460 cm^−1^ and shifting of bands around 1500 cm^−1^ and 1520 cm^−1^. Time constants of (2.0 ± 0.5) ps and (18 ± 3) ps show dispersive features around 1500 cm^−1^ and 1520 cm^−1^ reflecting cooling, narrowing, and spectral shifts of these bands. The spectral shifts of the zero-crossings are presented in Fig. S2.^50^ Since vibrational dynamics are directly connected to its electronic dynamics, common decay times describe the same dynamics. Thus, the DAS with decay time of (80 ± 8) ps displays the decay of the singlet excited state (positive signals) and the rise of the triplet absorption (negative signals). We obtain the same results upon analyzing the lifetime map of the vibrational data (presented in Fig. S3).^50^

**FIG. 9.**
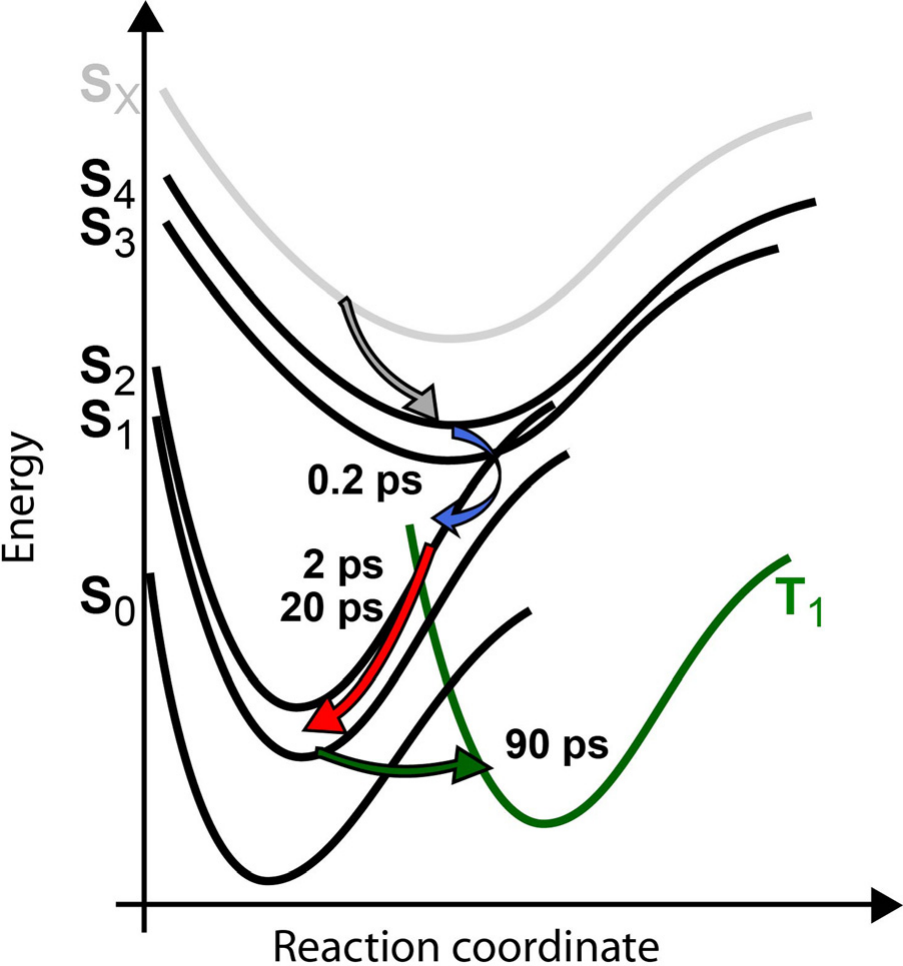
Sketch of the proposed photoreaction pathways. Upon excitation, a fast relaxation to the Q band (S_1_, S_2_) with a time constant of 250 fs occurs, followed by energy redistribution processes with 2 ps and 20 ps. The complete excited population is transferred to the triplet state with a time constant of 95 ps.

In summary, we propose the following photoreaction scheme presented in Fig. 9. After photoexcitation, the internal conversion in the Soret band is ultrafast, and energy relaxation into the Q band occurs with a time constant of 250 fs. In the Q band, energy relaxation processes on the picosecond time scale take place (see Fig. 9). With a time constant of 95 ps all population is transferred from the singlet excited state to the triplet state. We assume that this triplet state is the T_1_ state, because we do not detect further relaxation processes on a time scale of 300 ps, but we cannot exclude that the populated triplet state is an excited triplet state. We conclude that 100% of the excited state population is transferred to the triplet state, since we do not observe any bleaching recovery in the 95 ps time scale, and the stimulated emission signal completely vanishes with this time constant.

## III. CONCLUSION

We present the first comprehensive analysis of the photoreaction dynamics of a brominated corrole Al(tpfc-Br_8_)(py)_2_ by combining electronic and vibrational dynamics for the first time. We measured a very fast Soret to Q band transition with a time constant of 250 fs, which is fast compared to non-brominated Al(tpfc)py with a time constant of 500 fs,^34^ and other corrole systems.^32,34,45^ The energy redistribution processes occur on a picosecond time scale, similar to other corrole systems.^32,45^

Nevertheless, electronic absorption bands overlap significantly and thus prevent a clear assignment of triplet dynamics. We identified vibrational triplet marker bands at 1480 cm^−1^ and 1506 cm^−1^ as ideally suited for characterization of triplet formation and dynamics. In Al(tpfc-Br_8_)(py)_2_ triplet generation takes place with a time constant of 95 ps, significantly faster than intersystem crossing rates reported for free-base corroles and their germanium and phosphorus complexes,^32,45^ and with an extraordinarily high yield of about 100%. This exceptional high triplet quantum yield can be explained by the bromine atoms introducing a strong spin-orbit coupling. Hence, Al(tpfc-Br_8_)(py)_2_ is a very promising candidate as a photosensitizer with an expected high singlet oxygen yield. The identification of vibrational marker bands for separation of singlet and triplet dynamic opens new strategies to investigate the triplet formation in corroles and related molecules.

## IV. METHODS

Femtosecond laser pulses were generated starting from a fundamental femtosecond laser pulse delivered by a 1 kHz Ti:Sa laser system (Coherent Legend USP, 80 fs pulses at 800 nm). The fundamental beam was split into two parts for pump and probe pulse generation. The pump pulses were generated by second harmonic generation in a BBO crystal with a pulse energy of 0.25 *μ*J.

Angle balanced femtosecond polarization resolved VIS pump–IR probe measurements were applied as described elsewhere.^46,47^ In short, the mid-IR probe beam is generated by a difference frequency mixing step from near-infrared signal and idler pulses generated by 800 nm fs pulses in a BBO crystal. Two reflections of the fs mid-IR pulse are taken as probe beams with different polarizations used at the same time in the same sample volume to detect absorbance changes. The system response was about 350 fs with pump focus of about 200 *μ*m and probe focus of about 150 *μ*m. Absorbance changes with mid-IR polarizations parallel (A_pa_) and perpendicular (A_pe_) to the VIS pump beam polarization were detected. Isotropic absorbance changes (A_iso_) were calculated by A_iso_ = (A_pa_ + 2 A_pe_)/3. Here, we presented only isotropic data. In the IR dataset, we observed a rising featureless background with a delay time. This background is subtracted in the presented data. The background is probably induced by long-lasting triplet generation.

For isotropic Vis pump–supercontinuum probe measurements, we used a sapphire white light supercontinuum with polarization angle between both beams set to the magic angle (54.7°). Both beams were focused into the sample cell by a curved mirror. Behind the sample, a filter (HR-800 mirror) was used to suppress the fundamental in the supercontinuum, and the beam was focused into a prism-spectrometer (Stresing GmbH) equipped with a 512 pixel InGaAs sensor. Every second pump beam was blocked by a chopper to record excited and not excited sample volumes alternatively.^48^ The sample was moved perpendicular to the beam direction to minimize reexcitation. The system response was better than 100 fs (typically ∼80 fs). The Al(tpfc-Br_8_)(py)_2_ was synthesized as reported previously.^5,49^ Al(tpfc-Br_8_)(py)_2_ samples of about 0.008 mol/l were prepared with a maximal absorption of about 1 OD in the Q-band at 640 nm in d_8_-toluene.
